# The interactive effects of pre-pregnancy body mass index, thyroid function, and blood lipid levels on the risk of gestational diabetes mellitus: a crossover analysis

**DOI:** 10.1186/s12884-022-04908-4

**Published:** 2022-07-20

**Authors:** Ying Wang, Sha Lu, Xianrong Xu, Lijun Zhang, Jun Yang, Wensheng Hu

**Affiliations:** 1 Department of Nutrition and Toxicology, Hangzhou Normal University School of Public Health, Hangzhou, China; 2 Department of Obstetrics, The Affiliated Hangzhou Women’s Hospital, Hangzhou Normal University, Hangzhou, China; 3 Zhejiang Provincial Research Center for the Diagnosis and Treatment of Uterine Cancer, The Affiliated Women’s Hospital, Zhejiang University School of Medicine, Hangzhou, China

**Keywords:** Pre-pregnancy body mass index, Thyroid function, FT3/FT4 ratio, Blood lipid, Interaction, Gestational diabetes mellitus

## Abstract

**Background:**

Studies have demonstrated the associations between pre-pregnancy obesity, thyroid dysfunction, dyslipidemia, and increased risk of gestational diabetes mellitus (GDM) in pregnant women. This study was designed to investigate whether and to what extent, the interactions between these factors contribute to the risk of GDM.

**Methods:**

A case–control study of 232 GDM cases and 696 controls was conducted among pregnant women from Hangzhou, China. Multiple logistic regression analysis was applied to identify independent risk factors of GDM. Crossover analysis was performed to assess the interactive effects of pre-pregnancy body mass index (pBMI), thyroid hormones, and blood lipid profiles on the risk of GDM. The indexes including attributable proportion (AP) to the interaction and the relative excess risk due to interaction (RERI) were calculated.

**Results:**

Chinese pregnant women with pBMI > 23 kg/m^2^ (adjusted: OR = 4.162, *p* < 0.001), high triglyceride levels (> 2.30 mmol/L) (adjusted: OR = 1.735, *p* < 0.001), and the free triiodothyronine/free thyroxine (FT3/FT4) ratio ≥ 0.502 (OR = 4.162, *p* < 0.001) have significantly increased risk of GDM. Crossover analysis indicated that there were significant interactions between pre-pregnancy overweight/obesity and FT3/FT4 ≥ 0.502 (AP = 0.550, *p* < 0.001; RERI = 7.586, *p* = 0.009), high TG levels and FT3/FT4 ≥ 0.502 (AP = 0.348, 95%CI = 0.081–0.614, *P* = 0.010; RERI = 2.021, 95%CI = 0.064–3.978, *p* = 0.043) on the risk of GDM.

**Conclusion:**

The interactions between pBMI and FT3/FT4 ratio, TG level and FT3/FT4 ratio may have significant impacts on the risk of GDM in pregnant women. Such findings may help improve our understanding of the pathogenesis of GDM as well as develop comprehensive strategies for the management of GDM.

## Background

Gestational diabetes mellitus (GDM) is a common and growing antenatal disease that affects about 9%-25% of pregnant women worldwide [1]. It is likely to be associated with a variety of adverse birth outcomes such as macrosomia, fetal growth restriction, fetal distress, preterm birth, and is accompanied by a high incidence of cesarean delivery, obstructed shoulder delivery, and postpartum hemorrhage [2]. It poses a serious threat to the health of mothers and their offspring and has become a major public health problem [3]. Data from the International Diabetes Federation (IDF) showed that the global incidence of GDM in 2021 was about 14.0%, with the highest prevalence of GDM in Middle East and North Africa (27.6%) and South-East Asia (20.8%), followed by Western Pacific (14.7%) and Africa (14.2%) [4]. A meta-analysis investigating the prevalence of GDM in East and Southeast Asia showed that the prevalence of GDM in China was 11.91%, much higher than that of Japan, Korea, and Thailand [5]. Therefore, the prevention and treatment of GDM, as well as identifying causal risk factors, remain a great mission and momentous challenge in China.

Unfortunately, the pathogenesis of GDM is not yet fully understood, and there is evidence that both genetic and environmental factors are important determinants [6]. From the view of prevention, identifying modifiable risk factors for GDM is the first step toward primary prevention. Over the years some risk factors have been well established, such as pre-pregnancy obesity, endocrine factors, advanced age, and family history of diabetes [7, 8]. Among them, pre-pregnancy overweight or obesity (body mass index (BMI) ≥ 25 kg/m^2^) has been implicated as the most significant risk factor for GDM [9]. It has been shown that pre-pregnancy BMI (pBMI) is associated with increased risk of GDM in a dose–response manner [9, 10], and all three obesity phenotypes, namely general, central, and visceral obesity, are consistently associated with an increased risk of GDM in women [11]. Furthermore, there was evidence that pBMI may interact with other risk factors for GDM, including environmental [12], genetic [13], or behavioral risk factors [14], but the results were controversial. Interaction studies that assess the combined influence of pBMI and other risk factors may improve our understanding of GDM.

Maternal thyroid dysfunction [15] and dyslipidemia [16] are also important risk factors for GDM. Hypothyroxinemia, as well as subclinical and overt hypothyroidism, were observed to be significantly associated with the risk of GDM [17, 18]. Several cross-sectional studies have shown that the free triiodothyronine (FT3)/free thyroxine (FT4) ratio, a commonly used indicator for thyroid function, is associated with higher insulin resistance, glycated hemoglobin, fasting glucose, post-load glucose levels, and increased risk of GDM in pregnancy women [19, 20]. Dyslipidemia was also associated with insulin resistance and β-cell dysfunction in pregnant women [21] and was found to be an independent predictor for GDM [22]. Moreover, there was evidence that thyroid dysfunction may also be a risk factor for dyslipidemia [23, 24]. Therefore, the comprehensive effects of thyroid dysfunction and dyslipidemia on the risk of GDM need further investigation.

Although previous studies have confirmed that pBMI [25], FT3/FT4 ratio [20], and dyslipidemia [26] are all independent determinants of GDM, their interactive effects on the risk of GDM remain unclear. To address this issue, we performed a case–control study with crossover analysis to investigate the comprehensive effects of pBMI, thyroid hormone, and serum lipids on the risk of GDM in a Chinese population. The results from this study may help to advance our understanding of the pathogenesis of GDM.

## Subjects and methods

This study was approved by the Ethics Committee of Hangzhou Women’s Hospital and was carried out following the approved guidelines.

### Participants

Pregnant women who delivered at Hangzhou Women’s Hospital in Zhejiang Province of East China between March 2017 and July 2018 were included. The inclusion criteria were as follows: (1) natural conception, singleton pregnancy; (2) met the diagnostic criteria for GDM and no other pregnancy-related diseases; (3) not taking lipid/glycemic/thyroid function-related medications before and during pregnancy; (4) complete basic data and medical history. Exclusion criteria of pregnant women were: (1) multiple pregnancies; (2) diagnosis of type 1 or type 2 diabetes mellitus before or during pregnancy; (3) a history of severe complications including inherited metabolic diseases, cardiovascular disease, thyroid or liver dysfunction; (4) use of drugs that could affect lipid levels such as corticosteroids; and (5) miscarriage. We used the same exclusion criteria to identify patients without GDM as the control group, and case and control were matched for age (1:3 ratio). Finally, a total of 232 women with GDM and 696 healthy controls were enrolled in the study.

### Anthropometric and biochemical measurements

Demographic data were collected from the medical records by trained staff including the following basic information: (1) general characteristics (age, height, pre-pregnancy weight, gravidity, history of abortion.); (2) previous or current disease status, and autoimmune thyroid disease, medication use, and lifestyle habits. BMI was calculated by dividing the weight in kilograms by the height in square meters (kg/m^2^). We used the WHO expert group’s adjusted optimal BMI range for Asian populations for classification, with "non-overweight" defined as BMI < 23.00 kg/m^2^ and "overweight/obesity" defined as BMI ≥ 23.00 kg/m^2^ [27].

All pregnant women are routinely screened for GDM with a 75 g oral glucose tolerance test (OGTT) between 24 and 28 weeks of gestation. GDM was diagnosed if at least one of the following criteria were met: (a) fasting plasma glucose (FPG) ≥ 5.1 mmol/L (92 mg/dl); (b) 1 h plasma glucose ≥ 10.0 mmol/L (180 mg/dl); and (c) 2 h plasma glucose ≥ 8.5 mmol/L (153 mg/dl).

All women underwent a venous blood sample collection at 24–28 gestational weeks. Levels of free triiodothyronine (FT3), free thyroxine (FT4), and thyroid-stimulating hormone (TSH) were measured with a Beckman Coulter Unicel Dlx 800 Immunoluminescence Analyzer. According to the Guidelines for the Diagnosis and Treatment of Pregnancy and Postpartum thyroid Diseases, the normal reference range for TSH during pregnancy is 0.30 mIU/L ≤ TSH ≤ 3.00 mIU/L [28].

The triglyceride (TG), total cholesterol (TC), high-density lipoprotein cholesterol (HDL-C), and low-density lipoprotein cholesterol (LDL-C) levels were analyzed by a Beckman Coulter AU5800 automatic biochemistry analyzer. Since there are no diagnostic criteria for hyperlipidemia during pregnancy in China, we defined dyslipidemia in pregnant women under the Guidelines for the Prevention and Treatment of Dyslipidemia in Chinese Adults (Revised 2016) [29]: TG > 200 mg/dL (2.30 mmol/L), LDL-C > 160 mg/dL (4.10 mmol/L) or HDL-C < 40 mg/dL (1.30 mmol/L).

### Statistical analysis

All statistical analyses were performed using IBM SPSS Statistics for Windows version 20.0. Based on the literature search, age, gravity, and history of abortion were all associated with GDM, so they were adjusted for the analysis. Continuous data were expressed as mean ± standard deviation, while categorical data as number (*n*, %). Characteristics such as age, gravidity, BMI, FPG, and blood lipid profile at the first prenatal visit were compared between GDM and non‐GDM subjects. The non‐parametric and chi-square tests were used to compare the differences between groups. The restricted cubic spline analysis was performed using SAS 9.4 to identify the serum FT3/FT4 threshold corresponding to the risk of GDM. Multiple logistic regression was used to evaluate the associations between possible risk factors (thyroid hormones, blood lipid profiles, and pBMI) and the risk of GDM. The interactions between thyroid hormones, blood lipid profiles, and pBMI on the risk of GDM were evaluated by crossover analysis. Multiple comparisons were corrected using the Benjamini–Hochberg method. Statistical significance was defined as *P* < 0.05.

The crossover analysis is based on the epidemiological 2 × 4 cross-sectional analysis table, in which both factor 1 and factor 2 to be studied are represented as dichotomous variables, and the group in which neither factor is exposed is selected as the common reference group with OR = 1. OR values and corresponding 95% CI are calculated for each combination of exposure. Our study used a more detailed presentation of the crossover analysis table suggested by Knol and VanderWeele [30], and calculate the attributable proportion of interaction (AP) and the excess relative risk of interaction (RERI) based on the additive interaction model with the corresponding 95% CI and *P* values to present the interaction more clearly and comprehensively.

## Results

### General characteristics of the participants

Table 1 showed the selected participant characteristics among women with or without GDM. A total of 232 GDM patients and 696 healthy controls were enrolled for final analysis in this study. The average age of the participants was 29.9 ± 4.0 years. The proportion of primiparas and multiparas among the participants was 40.8% and 22.8%, respectively. The average pBMI of the participants was (22.27 ± 3.06) kg/m^2^ in the GDM group, which was significantly higher than that of the control group [(20.55 ± 1.95) kg/m^2^] (*P* < 0.001). The proportion of overweight/obesity among GDM patients was significantly greater than that of the subjects in the control group (43.1% *vs* 13.6%, *P* < 0.001). The average FT4 levels were significantly lower among GDM cases, whereas average FT3 levels and the FT3/FT4 ratio were significantly higher among GDM cases than those of controls (*P* < 0.001). TSH levels did not differ significantly between cases and controls. Compared with controls, GDM cases were more likely to have a lower level of TC, LDL-C, and HDL-C (*P* < 0.05), and a higher level of TG (*P* < 0.001). The proportion of high TG values was significantly higher in GDM cases than that of controls (*P* < 0.001). In contrast, the proportion of high TC, LDL-C, and HDL-C values showed no significant difference between the cases and controls (*P* > 0.05).

**Table 1:** Characteristics of participants with and without gestational diabetes mellitus

Characteristics	Total	GDM	Control	t/χ^2^	*P*
Mean ± SD / n (%)	(*n* = 928)	(*n *= 232)	(*n* = 696)		
Age, year	29.9 ± 4.0	30.3 ± 4.1	29.8 ± 3.9	-1.905	0.057
miscarriages	0.63 ± 0.90	0.68 ± 1.00	0.61 ± 0.86	-0.909	0.363
History of abortion					
No	537(57.9%)	125(53.9%)	412 (59.2%)	2.017	0.156
Yes	391(42.1%)	107(46.1%)	284 (40.8%)		
Gravidity	2.05 ± 1.16	2.12 ± 1.21	2.03 ± 1.15	-0.993	0.321
1	379(40.8%)	89(38.4%)	290 (41.7%)	0.786	0.375
≥ 2	549(9.2%)	143(61.6%)	406 (58.3%)		
Pre-pregnancy BMI, kg/m^2^	20.98 ± 2.39	22.27 ± 3.06	20.55 ± 1.95	-8.018	** < 0.001**
< 23.00	733(79.0%)	132(56.9%)	601(86.4%)	90.949	** < 0.001**
≥ 23.00	195(21.0%)	100(43.1%)	95(13.6%)		
TG,mmol/L	2.29 ± 0.88	2.56 ± 0.96	2.21 ± 0.83	-4.989	** < 0.001**
< 2.30	565(60.9%)	117(50.4%)	448(64.4%)	14.191	** < 0.001**
≥ 2.30	363(39.1%)	115(49.6%)	248(35.6%)		
TC, mmol/L	6.20 ± 1.06	6.03 ± 1.08	6.25 ± 1.05	2.769	**0.006**
< 6.20	482(51.9%)	131(56.5%)	351(50.4%)	2.538	0.111
≥ 6.20	446(48.1%)	101(43.5%)	345(49.6%)		
LDL-C, mmol/L	3.46 ± 0.82	3.31 ± 0.82	3.51 ± 0.81	3.307	**0.001**
< 4.10	737(79.4%)	191(82.3%)	546(78.4%)	1.602	0.206
≥ 4.10	191(20.6%)	41(17.7%)	150(21.6%)		
HDL-C, mmol/L	2.30 ± 0.46	2.15 ± 0.45	2.36 ± 0.45	6.268	** < 0.001**
TSH, mIU/L	1.95 ± 0.79	1.95 ± 0.74	1.96 ± 0.81	0.162	0.871
< 3.00	838(90.3%)	211(90.9%)	627(90.1%)	0.148	0.701
≥ 3.00	90(9.7%)	21(9.1%)	69(9.9%)		
FT3, pmol/L	4.28 ± 0.54	4.51 ± 0.84	4.20 ± 0.52	-7.697	** < 0.001**
FT4,pmol/L	8.41 ± 1.19	7.89 ± 1.12	8.58 ± 1.16	7.927	** < 0.001**
FT3/FT4	0.52 ± 0.10	0.58 ± 0.11	0.50 ± 0.09	-10.540	** < 0.001**

*P* value < 0.05 was considered statistically significant

*GDM* Gestational diabetes mellitus, *BMI* Body mass index, *TG* Triglyceride, *TC* Total cholesterol, *LDL-C* Low-density lipoprotein cholesterol, *HDL-C* High-density lipoprotein cholesterol, *TSH* Thyroid stimulating hormone, *FT3* Free triiodothyronine, *FT4 *Free thyroxine

### The association between FT3/FT4 ratio and the risk of GDM

**Fig. 1:**
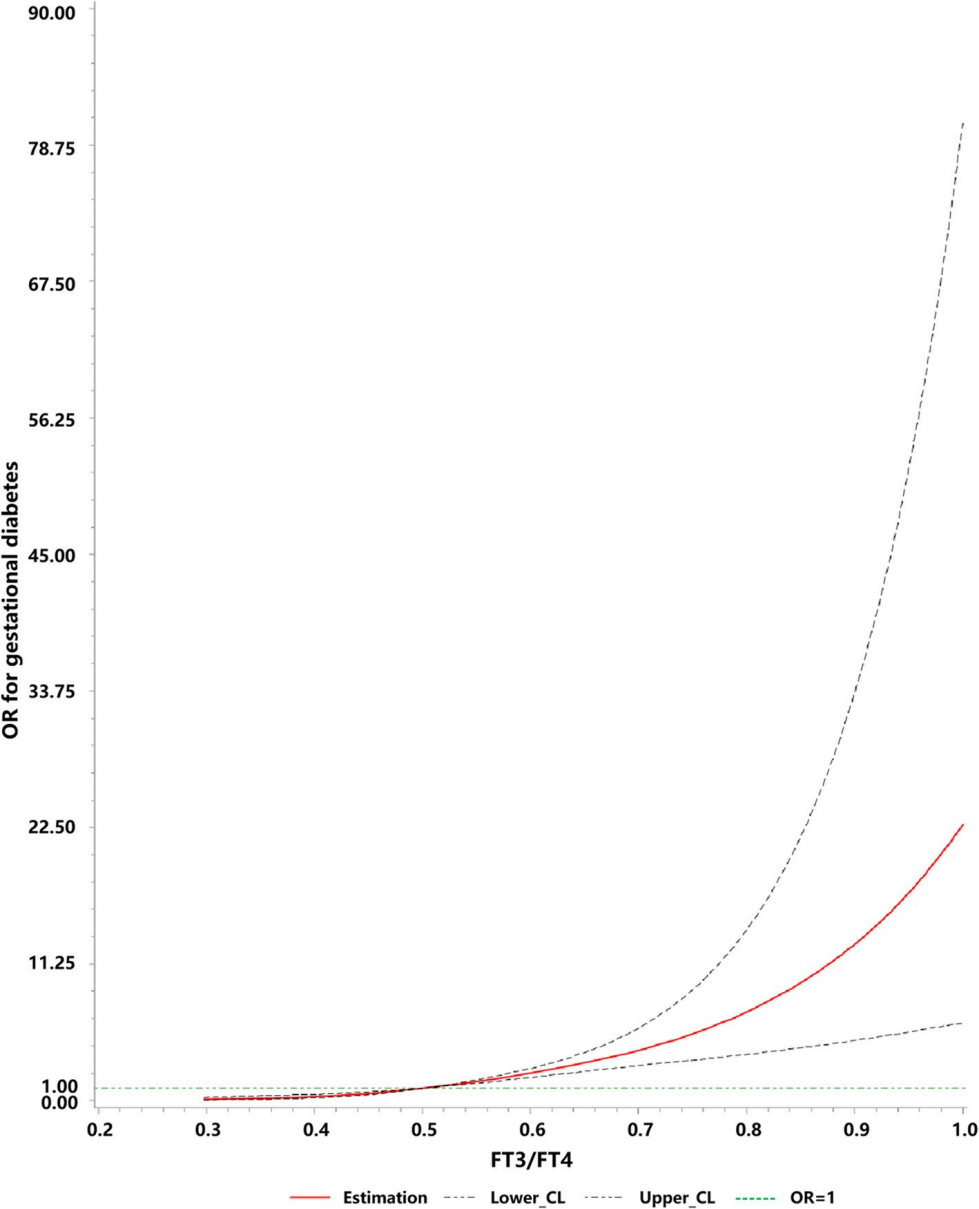
Dose–response relationship between FT3/FT4 ratio and the risk of gestational diabetes mellitus in pregnancy women. Adjusted for age, history of abortion, and gravidity. The range (min, max) of FT3/FT4 is: (0.297, 0.944)

To investigate the relationship between the FT3/FT4 ratio and the odds ratio (OR) of GDM, a spline curve analysis was performed (Fig. 1). The OR of GDM increased with the increment of FT3/FT4 ratio. The FT3/FT4 ratio ≥ 0.502 was confirmed to be the optimal cut-off value when the OR of FT3/FT4 ratio for GDM risk is equal to 1 (indicating no association). In other words, FT3/FT4 ≥ 0.502 is a risk factor for the development of GDM, while FT3/FT4 < 0.502, is “protective” against GDM.

The relationships between FT3/FT4, blood lipid profiles, and pBMI with GDM were assessed by univariate and multiple binary logistic regression, with or without adjustment for age, history of abortion, gravidity and pBMI. The FT3/FT4 ratio, pBMI, and TG levels were found to be significantly associated with the risk of GDM (Table 2). Compared with those with FT3/FT4 ratio < 0.502, pregnant women with FT3/FT4 ratio ≥ 0.502 had an increased risk of GDM (unadjusted OR = 4.248, 95%CI = 3.040–5.936, *p* < 0.001; adjusted OR = 3.459, 95%CI = 2.445–4.895, *p *< 0.001). Pre-pregnancy overweight/obesity women are more likely to have GDM compared to non-overweight women (unadjusted OR = 4.793, 95%CI = 3.418–6.721, *p* < 0.001; adjusted OR = 4.685, 95%CI = 3.329–6.594, *p* < 0.001). The risk of GDM in women with high TG level (> 2.30 mmol/L) during pregnancy was as 1.776 times as that in women with low TG level (≤ 2.30 mmol/L) (unadjusted: OR = 1.776, 95%CI = 1.315–2.398, *P* < 0.001; adjusted: OR = 1.566, 95%CI = 1.139–2.153, *p* = 0.006).

**Table 2:** Associations of thyroid function, blood lipid levels and pre-pregnancy BMI with risk of GDM

		Unadjusted OR (95%CI)	Unadjusted *P*	Adjusted OR (95% CI)	Adjusted *P*
pre-pregnancy BMI^a^	< 23.00 kg/m^2^	1.000		1.000	
	≥ 23.00 kg/m^2^	4.793 (3.418–6.721)	** < 0.001**	4.685 (3.329–6.594)	** < 0.001**
TG^b^	< 2.30 mmol/L	1.000		1.000	
	≥ 2.30 mmol/L	1.776 (1.315–2.398)	** < 0.001**	1.566 (1.139–2.153)	**0.006**
TC^b^	< 6.20 mmol/L	1.000		1.000	
	≥ 6.20 mmol/L	0.784 (0.582–1.058)	0.112	0.868 (0.633–1.191)	0.382
LDL-C^b^	< 4.10 mmol/L	1.000		1.000	
	≥ 4.10 mmol/L	0.781 (0.533–1.146)	0.206	0.818 (0.548–1.223)	0.328
TSH^b^	< 3.00 mIU/L	1.000		1.000	
	≥ 3.00 mIU/L	0.904 (0.542–1.510)	0.701	0.998 (0.580–1.718)	0.995
FT3/FT4^b^	< 0.502	1.000		1.000	
	≥ 0.502	4.248 (3.040–5.936)	** < 0.001**	3.459 (2.445–4.895)	** < 0.001**

^a^Adjusted for age, history of abortion and gravidity; ^b^Adjusted for age, history of abortion, gravidity and pBMI

*P* value < 0.05 was considered statistically significant

*BMI* Body mass index, *GDM* Gestational diabetes mellitus, *TG* Triglyceride, *TC* Total cholesterol, *LDL-C* Low-density lipoprotein cholesterol, *TSH* Thyroid stimulating hormone, *FT3* Free triiodothyronine, *FT4* Free thyroxine

### Interaction between FT3/FT4 ratio and pBMI on the risk of GDM

We further analyzed the interactions among FT3/FT4, pBMI, and blood lipid levels (TG) on the risk of GDM. The pairwise interaction model among the three was at the 2 × 2 level. All analyses were compared with or without the adjustment for age, history of abortion, and gravidity. Table 3 showed the interactive effect of FT3/FT4 and pBMI on the risk of GDM. After adjustment for age, history of abortion, and gravidity, compared with those who had a non-risk ratio of FT3/FT4 (< 0.502) and non-overweight, overweight, or obese women with a risk ratio of FT3/FT4 (≥ 0.502) had a significantly higher risk of GDM (OR = 13.799, 95%CI = 8.550–22.271, *p* < 0.001). Within the category of pBMI, pregnant women with a risk ratio of FT3/FT4 had a higher risk of GDM than those at non-risk ratio (OR = 3.689, 95%CI: 1.895–7.179, *p* < 0.001 for overweight/obesity women and OR = 3.491, 95%CI: 2.324–5.244, *p* < 0.001 for non-overweight women, respectively). Within the category of FT3/FT4 ratio, pregnant women with overweight/obesity had a significantly higher risk of GDM than those non-overweight pregnant women (OR = 4.117, 95%CI: 2.692–6.295*,* *p* < 0.001 for those with a risk ratio of FT3/FT4, and OR = 3.729, 95%CI: 1.934–7.190, *p* < 0.001 for those with non-risk FT3/FT4 ratio, respectively). A positive interaction was found between overweight/obesity and risk ratio of FT3/FT4 in the additive models (AP = 0.550, 95%CI = 0.322–0.777, *p* < 0.001; RERI = 7.586, 95%CI = 1.883–13.289, *p* = 0.009).

**Table 3:** Interaction between FT3/FT4 and pBMI for the risk of GDM*

FT3/FT4 ratio during pregnancy	Non-overweight (pBMI < 23.00)		overweight/obesity (pBMI ≥ 23.00)		OR (95%CI) for overweight /obesity within category of thyroid function	AP (95%CI)	RERI (95%CI)
	GDM/Control (n)	OR(95%CI)	GDM/Control (n)	OR (95%CI)			
Non-risk ratio (< 0.502)	40/363	1.000	17/41	3.729 (1.934–7.190) ***P* < 0.001**	3.729 (1.934–7.190) ***P* < 0.001**	0.550 (0.322–0.777) ***P* < 0.001**	7.586 (1.883–13.289) ***P* = 0.009**
Risk ratio (≥ 0.502)	92/238	3.491 (2.324–5.244) ***P* < 0.001**	83/54	13.79 (8.550–22.271) ***P* < 0.001**	4.117 (2.692–6.295) ***P* < 0.001**		
OR (95% CI) for risk ratio within category of pBMI		3.491 (2.324–5.244) ***P* < 0.001**		3.689 (1.895–7.179) ***P* < 0.001**			

^*^Adjusted for age, history of abortion and gravidity

*P* value < 0.05 was considered statistically significant

*pBMI* Prepregnancy body mass index, *GDM* Gestational diabetes mellitus, *FT3* Free triiodothyronine, *FT4* Free thyroxine, *RERI* Relative excess risk due to interaction, *AP* Attributable proportion to the interaction

### Interaction between FT3/FT4 ratio and blood lipid levels on the risk of GDM

Table 4 showed the effect of the interaction between FT3/FT4 and blood lipid levels for the risk of GDM. After adjustment for age, history of abortion, and gravidity, compared with those with low TG levels and the non-risk ratio of FT3/FT4, pregnant women with high TG levels and a risk ratio of FT3/FT4 had a significantly higher risk for GDM (OR = 5.812, 95%CI: 3.733–9.049, *p* < 0.001). Within the category of the risk ratio of FT3/FT4, pregnant women with high TG levels had a significantly higher risk of GDM (OR = 1.648, 95%CI = 1.127–2.410, *p* = 0.010). Within the category of TG levels, pregnant women with a risk ratio of FT3/FT4 had a higher risk of GDM than those with a non-risk ratio (OR = 4.603, 95%CI: 2.688–7.881, *p* < 0.001 for those with high TG levels, and OR = 3.551, 95%CI: 2.289–5.508, *p* < 0.001 for those with low TG levels). A positive interaction was found between high TG levels and risk ratio of FT3/FT4 in the additive models (AP = 0.348, 95%CI = 0.081–0.614, *p* = 0.010; RERI = 2.021, 95%CI = 0.064–3.978, *p* = 0.043).

**Table 4:** Interaction between FT3/FT4 and TG levels for the risk of GDM^*^

TG levels	Non-risk ratio (FT3/FT4 < 0.502)		Risk ratio (FT3/FT4 ≥ 0.502)		OR (95%CI) for risk ratio within category of blood lipid levels	AP (95%CI)	RERI (95%CI)
	GDM/Control (n)	OR (95%CI)	GDM/Control (n)	OR (95%CI)			
Low levels (< 2.30)	36/277	1.000	81/171	3.551 (2.289–5.508) ***P* < 0.001**	3.551 (2.289–5.508) ***P* < 0.001**	0.348 (0.081–0.614) ***P* = 0.010**	2.021 (0.064–3.978) ***P* = 0.043**
High levels (≥ 2.30)	21/127	1.241 (0.694–2.218) *P* = 0.466	94/121	5.812 (3.733–9.049) ***P* < 0.001**	4.603 (2.688–7.881) ***P* < 0.001**		
OR (95% CI) for high TG levels within category of thyroid function		1.241 (0.694–2.218) *P* = 0.466		1.648 (1.127–2.410) ***P* = 0.010**			

^*^Adjusted for age, history of abortion and gravidity

*P* value < 0.05 was considered statistically significant

*GDM* Gestational diabetes mellitus, *TG* Triglyceride, *FT3* Free triiodothyronine, *FT4* Free thyroxine, *RERI* Relative excess risk due to interaction, *AP* Attributable proportion to the interaction

### Interaction between pBMI and blood lipid levels on the risk of GDM

Table 5 showed the interactive effects between pBMI and blood lipid levels for the risk of GDM. After adjustment for age, history of abortion, and gravidity, compared with non-overweight women with a non-risk ratio of FT3/FT4, pregnant women with overweight/obesity and risk ratio of FT3/FT4 had a significantly higher risk of GDM (OR = 6.751, 95%CI = 4.203–10.843, *p* < 0.001). Within the category of non-overweight, women with high TG levels had a significantly higher risk of GDM (OR = 1.610, 95%CI = 1.097–2.363, *p* = 0.015). Within the category of TG levels, pregnant women with overweight/obesity had a significantly higher risk of GDM than those non-overweight pregnant women (OR = 4.771, 95%CI = 2.961–7.688, *p* < 0.001 for women with low TG levels, and OR = 4.164, 95%CI = 2.540–6.826, *p* < 0.001 for women with high TG levels). The interaction between pBMI and TG levels was not significant in the additive models (*p*-value for both RERI and AP was > 0.05).

**Table 5:** Interaction between pBMI and TG levels for the risk of GDM^*^

TG levels	Non-overweight (pBMI < 23.00)		Overweight (pBMI ≥ 23.00)		OR (95%CI) for risk ratio within category of blood lipid levels	AP (95%CI)	RERI (95%CI)
	GDM/Control (n)	OR (95%CI)	GDM/Control (n)	OR (95%CI)			
Low levels (< 2.30)	72/397	1.000	60/204	4.771 (2.961–7.688) ***P* < 0.001**	4.771 (2.961–7.688) ***P* < 0.001**	0.203 (-0.229–0.635) *P* = 0.357	1.370 (-1.958–4.698) *P* = 0.420
High levels (≥ 2.30)	45/51	1.610 (1.097–2.363) ***P* = 0.015**	55/44	6.751 (4.203–10.843) ***P* < 0.001**	4.164 (2.540–6.826) ***P* < 0.001**		
OR (95% CI) for high TG levels within category of pBMI		1.610 (1.097–2.363) ***P* = 0.015**		1.437 (0.815–2.532) *P* = 0.210			

^*^Adjusted for age, history of abortion and gravidity

*P* value < 0.05 was considered statistically significant

*pBMI* Prepregnancy body mass index, *GDM* Gestational diabetes mellitus, *TG* Triglyceride, *RERI* Relative excess risk due to interaction, *AP* Attributable proportion to the interaction

## Discussion

In line with previous studies [31, 32], in this case–control study, we found overweight and obesity before pregnancy, high serum TG levels, and FT3/FT4 ratio were correlated with increased risk of GDM. Moreover, we observed a significant interactive effect between pBMI and FT3/FT4 ratio on the risk of GDM. In additive models incorporating pBMI and FT3/FT4 ratio, there was a statistically significant positive interaction between pre-pregnancy overweight/obesity and FT3/FT4 risk ratio. These results indicated that thyroid hormone levels modified the effect of pre-pregnancy obesity on the risk of GDM, and the risk of GDM attributable to the interaction is as high as 55.0%. Furthermore, GDM attributed to the interaction was 7.586 times higher than other factors. Therefore, better control of pBMI might reduce the risk of those women with abnormal FT3 or FT4 levels during pregnancy to develop GDM. To our best knowledge, this study is one of the first to examine the interaction between pBMI and thyroid function on the risk of GDM.

Obesity is the most important risk factor for GDM, contributing to 46.3% of the overall population-attributable fraction risk in pregnant women [33]. However, it has been demonstrated that the BMI of those Asian women with high risk for GDM is much lower than the current WHO standard for overweight (BMI ≥ 25 kg/m^2^) [34]. Therefore, in the current study, a cut-off value of 23 kg/m^2^, which has been shown to be a better fit for Asian population, was applied. Our results indicated that BMI ≥ 23 kg/m^2^ indeed is a risk factor for GDM, suggesting that the standard for pre-pregnancy weight should be lowered. FT3/FT4 ratio, a commonly used proxy for peripheral deiodinase activity [35], is elevated and associated with poorer metabolic parameters such as unfavorable lipids profiles, blood pressure, and insulin resistance [36, 37]. Though the underlying mechanisms of the interaction have not been fully elucidated, the increment of FT3/FT4 ratio was demonstrated to be associated with higher fasting glucose, glycated hemoglobin concentrations, and insulin resistance [38, 39]. Moreover, elevated FT3/FT4 ratio may reflect the presence of metabolic syndrome components and high levels of adipose-related inflammatory markers in the participants [40]. Therefore, pregnant women with both overweight/obesity and elevated FT3/FT4 ratio were prone to have a higher risk of GDM. Since overweight/obesity may stimulate the activity of deiodinase and lead to reduced FT4 level, an elevated FT3/FT4 ratio may increase the risk of developing GDM as a mediating effect of overweight/obesity, which provides a new direction for future study that focuses on the mediating effect of different factors on the development of GDM [20].

The identification of a cut-off value for FT3/FT4 is an important finding of our study. In clinical practice, we might be able to use this value to identified those women with higher risk for GDM. On the other hand, it is not clear whether our findings can be directly applied to other ethnic groups, because it is well-known that GDM occurs in different ethnic groups with varying incidence. Furthermore, FT3/FT4 ratio changes with BMI and age  [41]. Finally, the shift in the ratio may be a para phenomenon, and not associated with causality. Consequently, future experimental and randomized controlled studies are needed to confirm our findings and to better understand the biological mechanisms underlying this interaction.

In addition, a significant interaction between high TG levels and FT3/FT4 ratio (≥ 0.502) on the risk of GDM in pregnant women was also identified in our study. The result indicated that 34.8% of the GDM risk could be attributed to the presence of interaction between thyroid hormone and TG levels in pregnant women. Furthermore, GDM attributed to the interaction was 2.021 times higher than other factors. Dyslipidemia during pregnancy, especially hypertriglyceridemia, was closely associated with a greater risk of GDM [21, 42], and the possible mechanisms including impaired insulin action, β-cell function, and nitric oxide signaling [21, 43]. As shown by a previous study, FT3/FT4 ratio was positively associated with multiple risk factors for GDM, including homeostasis model assessment of insulin resistance (HOMA-IR), waist circumference, triglyceride levels, fasting blood glucose, and systolic blood pressure [44]. Therefore, the co-presence of high TG and FT3/FT4 ratio may synergistically increase the risk of GDM. Furthermore, 67.9% of the association between higher FT3/FT4 ratio and increased risk of GDM might be mediated through the composite effect of some lipids [19]. As it is well known that thyroid hormones can affect serum lipid levels [45], we speculated that the interactive effect of higher TG level and FT3/FT4 ratio on the risk of GDM may be, at least partly, mediated through the regulation of serum lipids. Nonetheless, it is also possible that higher pBMI could lead to the positive interaction between TG and FT3/FT4. The reason may be that overweight/obesity leads to abnormal metabolism, resulting in high TG and thyroid dysfunction, and the interaction of these factors further promotes the development of GDM.

There are several advantages to our study. First, we explored the effects of pBMI, thyroid hormones, and serum lipids during pregnancy and their interactions on the risk of GDM. To our knowledge, it is the first study to explore these interactions by using crossover methodology. Second, we chose FT3/FT4 ratio as an indicator of thyroid hormone status, which has been demonstrated to be a stable and better predictor of insulin resistance and metabolic syndrome [46]. Third, we used the interaction presentation table recommended by Knol and VanderWeele [30] to report OR value, AP values, RERI interaction indices, and 95% CI. This analysis could provide not only the respective main effects of different risk factors but also the interactive values of the two factors on diseases based on different models. Fourth, the basic data of pregnant women including age, pre-pregnancy weight, pregnancy, and maternal delivery, as well as various clinical biochemical data such as serum thyroid hormone and lipid levels were all obtained from the hospital medical record files, thus reducing the errors caused by self-reporting.

Some limitations should also be noticed in our study. First, although some potential confounding factors were controlled statistically, we cannot rule out the residual confounding effects that may affect some of our findings. For example, information of some known risk factors for GDM, such as family history of diabetes, smoking, gestational weight gain, and inflammation factors, was not obtained in this study. Thus, interpreting the results of the present study should be done with caution. Second, the blood sample was taken at diagnosis, and for prediction earlier samples for FT3, FT4, and lipids should be superior. Third, due to the nature of the case–control study, the specific mechanisms and the causal associations could not be explored. Therefore, a larger sample size, well-designed cohort studies are warranted for further exploration and verification. In addition, our study is a single-center study in China, with a relatively small number of cases, which may produce some bias in the statistical results, and further studies are needed to examine the reproducibility in other settings.

## Conclusion

Taken together, our study demonstrated that pBMI, thyroid function during pregnancy (FT3/FT4), and lipid levels (TG) were all associated with increased risk of GDM. The interactions between pBMI and FT3/FT4 ratio, TG level and FT3/FT4 ratio may have significant impacts on the risk of GDM in pregnant women. Therefore, we should carefully monitor pBMI, thyroid function and lipid changes during pregnancy, so as to develop targeted interventions to reduce the risk of developing GDM in future clinical practice.

## Data Availability

The data that support the findings of this study are available from Hangzhou Women's Hospital but restrictions apply to the availability of these data, which were used under license for the current study, and so are not publicly available. Data are however available from the authors upon reasonable request and with permission of Hangzhou Women's Hospital.

## Abbreviations

GDM: Gestational diabetes mellitus
pBMI: Pre-pregnancy body mass index
FT3: Free triiodothyronine
FT4: Free thyroxine
TSH: Thyroid-stimulating hormone
TG: Triglyceride (TG)
TC: Total cholesterol
HDL-C: High-density lipoprotein cholesterol
LDL-C: Low-density lipoprotein cholesterol
AP: Attributable proportion to the interaction
RERI: The relative excess risk due to interaction
